# One-step solvothermal deposition of ZnO nanorod arrays on a wood surface for robust superamphiphobic performance and superior ultraviolet resistance

**DOI:** 10.1038/srep35505

**Published:** 2016-10-24

**Authors:** Qiufang Yao, Chao Wang, Bitao Fan, Hanwei Wang, Qingfeng Sun, Chunde Jin, Hong Zhang

**Affiliations:** 1 School of Engineering, Zhejiang A & F University, Hangzhou, Zhejiang Province, 311300, PR China; 2 Key Laboratory of Wood Science and Technology, Zhejiang Province, 311300, PR China

## Abstract

In the present paper, uniformly large-scale wurtzite-structured ZnO nanorod arrays (ZNAs) were deposited onto a wood surface through a one-step solvothermal method. The as-prepared samples were characterized by X-ray diffraction (XRD), field-emission scanning electron microscopy (FE-SEM), transmission electron microscopy (TEM), Fourier transform infrared spectroscopy (FTIR), thermogravimetry (TG), and differential thermal analysis (DTA). ZNAs with a diameter of approximately 85 nm and a length of approximately 1.5 μm were chemically bonded onto the wood surface through hydrogen bonds. The superamphiphobic performance and ultraviolet resistance were measured and evaluated by water or oil contact angles (WCA or OCA) and roll-off angles, sand abrasion tests and an artificially accelerated ageing test. The results show that the ZNA-treated wood demonstrates a robust superamphiphobic performance under mechanical impact, corrosive liquids, intermittent and transpositional temperatures, and water spray. Additionally, the as-prepared wood sample shows superior ultraviolet resistance.

Wood or wood-based products are preferred materials, mainly due to their superior physical properties and warm appearance, which distinguish them in many areas from other competitive materials such as concrete, metals and plastics^1^,^2^,^3^. However, when exposed to outdoor conditions, wood surfaces lose their efficacy through discolouration, clouding, roughening and checking, especially when faced with enormous threats and challenges such as acid rain, high-temperature–humidity conditions or ultraviolet irradiation^4^,^5^. Therefore, developing a shield for wooden surfaces might be an efficient pathway for preventing the loss of appearance in outdoor services.

ZnO is one of the most important functional materials, used in areas from fundamental research to industrial applications because of its unique photo-catalytic, electrical, electronic, optical, dermatological, antibacterial and ultraviolet-resistant properties^6^,^7^. Furthermore, ZnO is intrinsically active due to the abundant presence of hydroxyl groups on its surface, which can be designed to effectively adhere to the surface of wood^8^,^9^,^10^. Our previous studies have reported that wood surfaces can be readily applied with a ZnO coating through a hydrothermal method^8^,^10^. In fact, many different methods for synthesizing ZnO nanorod arrays deposited on wood surfaces have been published, such as hydrothermal synthesis^10^, wet chemical approach^9^, chemical vapour deposition^11^ and liquid-phase deposition^12^. However, these preparative methods are generally complicated and include at least two or three steps for the formation of ZnO nanorods on the wood surface (e.g., the first step is preplanting ZnO nanoseeds on the wood surface; the second step is growing ZnO nanorods by the corresponding method). Further, superhydrophobic ZnO-treated wood has been realized by the use of low-surface-energy liquids. For widespread use of ZnO nanorod arrays on wood substrates, a readily manipulated and highly simple process should be developed.

The present work describes the deposition of ZnO nanorod arrays on a wood surface through a simple, one-step solvothermal method. The possible mechanisms of both formation of ZnO nanorod arrays and their deposition on the wood surface through the one-step solvothermal method are discussed. The prepared ZNA-treated wood shows a robust superamphiphobic performance and superior ultraviolet resistance, according to measurements from sand abrasion tests and artificially accelerated ageing tests.

## Results and Discussion

**Figure 1:**
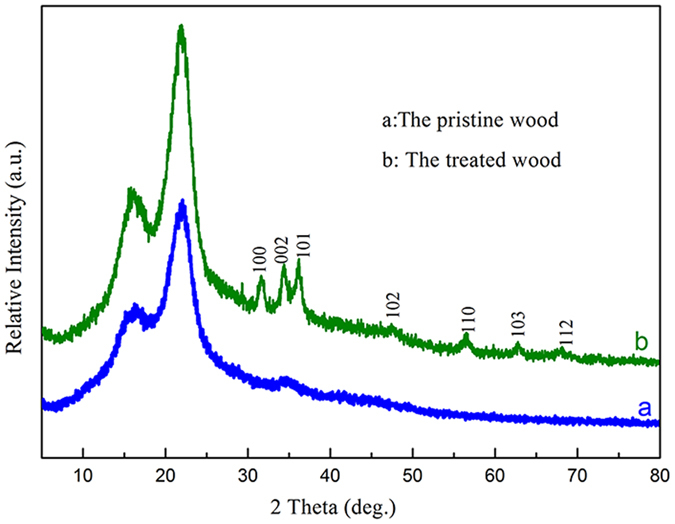
XRD patterns of (**a**) the pristine wood and (**b**) the ZNA-treated wood.

Figure 1 shows the XRD patterns of the pristine wood and the ZNA-treated wood, respectively. In Fig. 1a, the diffraction peaks at approximately 15° and 22° correspond to the crystalline region of the cellulose of pristine wood^13^,^14^. However, some new, strong diffraction peaks were observed in Fig. 1b, and these are well-indexed to the standard diffraction pattern of hexagonal-phase ZnO (JCPDS card No. 36–1451)^15^,^16^. This suggests that wurtzite-structured ZnO covers the wood surface. No characteristic peaks from other phases of ZnO were found, suggesting that the ZNAs had high phase-purity and a pure wurtzite crystal structure.

**Figure 2:**
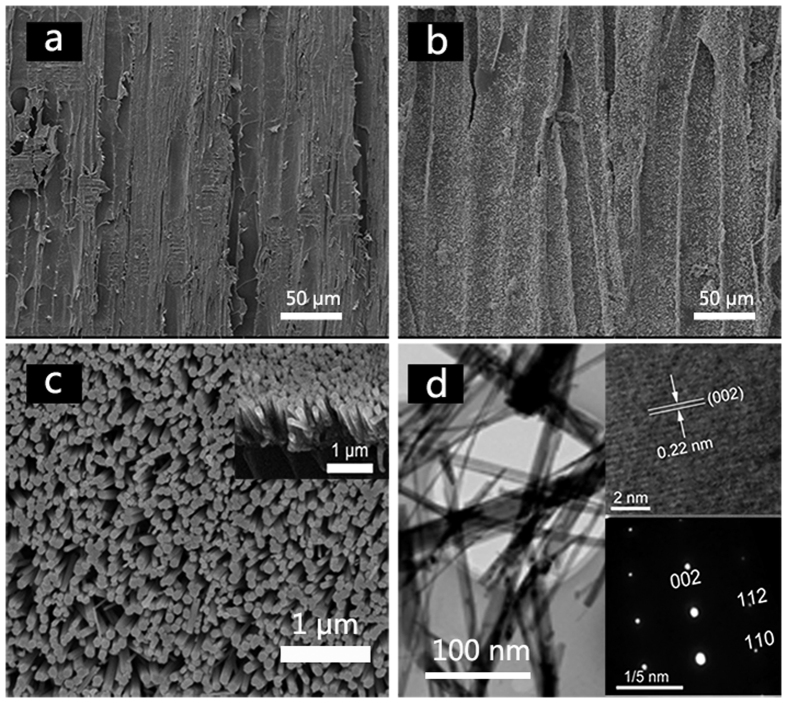
SEM images of (**a**) the surface of the pristine wood, (**b**) the surface of the ZNA-treated wood with low-magnification, (**c**) the surface of the ZNA-treated wood with high-magnification, the inset image is the cross section of ZNAs, (**d**) TEM images of ZNAs peeled off from the wood surface. The inset images were the corresponding HRTEM image (top right corner) and SAED pattern (right bottom).

The surface morphologies of the pristine wood and the ZNA-treated wood are exhibited in Fig. 2. In Fig. 2a, pits and fibrils can be clearly observed on the pristine wood surface. However, after the one-step solvothermal treatment, the wood surface was uniformly covered by a dense and even film, as shown in Fig. 2b. In the magnified SEM image, presented in Fig. 2c, a dense film was clearly formed by ZnO nanorod arrays. These grew vertically on the wood substrate, with an almost identical height. The cross-section view in the inset of Fig. 2c indicates that the ZNAs had a diameter of approximately 85 nm and a length of approximately 1.5 μm. The fine features of the ZNAs were further investigated by the TEM technique. A typical TEM image, shown in Fig. 2d, shows ZnO nanorods with a rod-like shape, peeled off from the treated wood sample by strong ultrasonic treatment at 1800 W. The HRTEM image of ZnO nanorods, in the top right corner of Fig. 2d, exhibits well-ordered crystalline planes with a lattice spacing of 0.22 nm, which is consistent with the spacing of the (0 0 2) planes of wurtzite-structured ZnO. Additionally, The SAED pattern in the bottom right of Fig. 2d confirmed the single crystallinity, with the *c*-axis being the preferred orientation of the obtained ZnO nanorods. Therefore, the results of HRTEM and SAED demonstrated that ZNAs covering the treated wood surface grew along the *c* direction, which is consistent with the XRD results shown in Fig. 1b.

**Figure 3:**
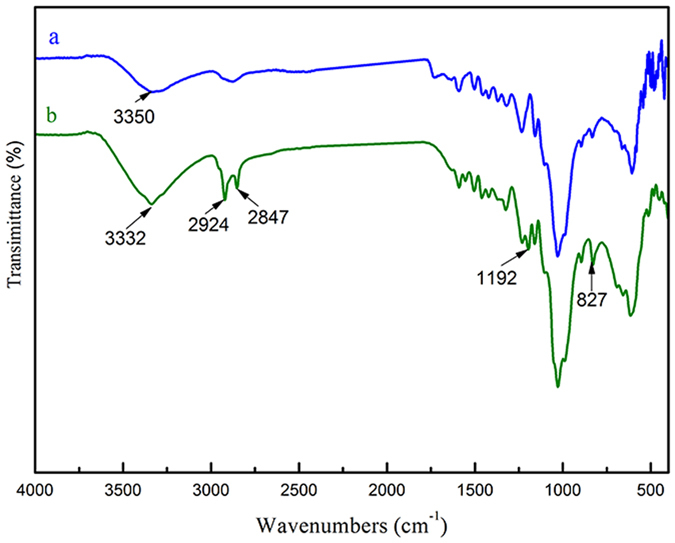
FTIR spectra of (**a**) the pristine wood and (**b**) the ZNA-treated wood.

Figure 3 shows the FTIR spectra of the pristine and ZNA-treated wood samples. The peaks at 3330–3350 cm^−1^ were assigned to stretching vibrations of hydroxyl groups on the wood surface, which shifted to lower wavenumbers, indicating a strong interaction between the hydroxyl groups of the wood surface and ZnO nanorod arrays through hydrogen bonds^17^. This strong interaction led to the deposition of ZNAS vertically implanted onto the wood surface. The two strong absorption peaks at 2919 cm^−1^ and 2852 cm^−1^ were ascribed to the asymmetrical stretching vibrations of −CH_3_ and −CH_2_^18^. The presence of the shoulder peaks reveals that long-chain alkyl groups were constructed on the ZNA-treated wood surface, which might be responsible for the superamphiphobic performance. The absorption peak at 1192 cm^−1^ was attributed to the stretching vibration of C-F incorporated into the ZnO surface^9^. The absorption peak at approximately 827 cm^−1^ was assigned to the bending vibration of Si-O, which was from the alcoholysate of FAS formed under solvothermal conditions. In addition, the absorption band observed at approximately 500 cm^−1^ was attributed to the ZnO stretching vibrations^8^. The analysis of FTIR spectra of the pristine wood and ZNA-treated wood suggests chemical interactions between the wood surface and ZNAs and the existence of potential superamphiphobic performance, both of which were obtained through the one-step solvothermal method.

**Figure 4:**
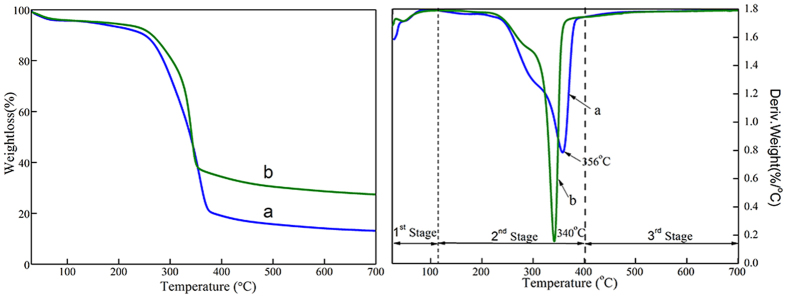
TG-DTA curves of (**a**) the pristine wood and (**b**) the ZNA-treated wood.

Figure 4 shows the thermogravimetric and differential thermogravimetric analysis (TG–DTG) curves of the pristine wood (a) and ZNA-treated wood (b) under a nitrogen atmosphere. In the first stage (20 °C to 105 °C), a small weight loss (3.93%) was observed in both specimens, which was attributed to the evaporation of moisture from the surfaces of the samples. In the second stage (105 °C to 400 °C), the main weight loss had already occurred due to the pyrolysis of wood cellulose and hemicellulose, and the maximum pyrolysis rate occurred at 356 °C and 340 °C, with weight losses reaching 65.04% for the pristine wood and 50.63% for ZNA-treated wood, respectively. In the third stage (400 °C to 700 °C), the mass percentages of pyrolysis residue were approximately 4.9 and 7.5 mass% for the pristine wood and ZNA-treated wood, respectively.

In the one-step solvothermal process of ZNA deposition onto the wood surface, many more complex combinations of zinc acetate dehydrate with sodium hydroxide might be possible, which occur in the presence of CTAB and facilitate a good alkaline environment. Initially, when zinc acetate dehydrate was dissolved into 95% ethanol solution and heated in a sealed surrounding, Zn(OH)_2_ was formed and continued to develop, then disintegrated and led to the development of crystal growth configurations^19^ (Eq. 1). As the process advanced in the alkaline atmosphere, (Zn[OH]_4_) formed the units of crystal growth and development^20^,^21^ (Eqs 2, 3). Then, (Zn[OH]_4_)^2−^ seemed to mature, leading to the formation of ZnO nanocrystals^22^ (Eq. 4). Simultaneously, the complexes of CTAB and Zn(OH)_2_ adsorbed on the ZnO surfaces led to the elongation of the c-axis, which was actively elongated towards the 0001 facet due to electrostatic forces of attraction^22^,^23^,^24^,^25^,^26^ (Eqs 5, 6, 7, 8). Finally, this anisotropic growth resulted in the formation of rod-like structures and slightly broadened the width of the transverse direction, thus giving rise to the assembly of rod-shaped ZnO arrays.


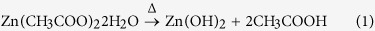



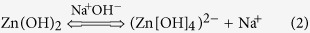



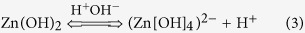



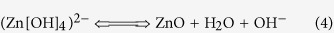



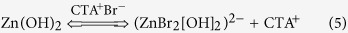



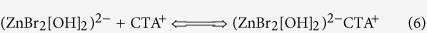



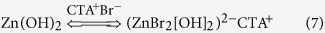



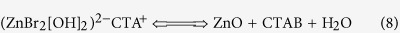


**Figure 5:**
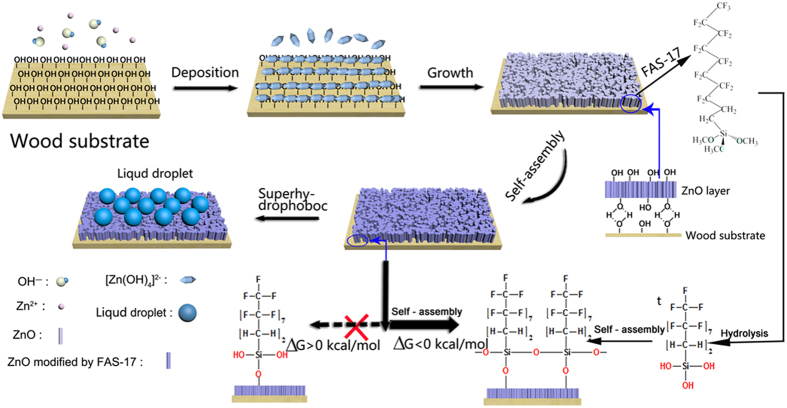
Schematic illustration of the preparation of the superamphiphobic ZNA-treated wood.

On the basis of the results mentioned above, a schematic illustration of the creation and implantation of ZnO nanorod arrays onto a wood surface through one-step solvothermal method is proposed in Fig. 5. First, when the wood substrate is immersed into the mixed solution containing zinc ions and hydroxyl ions (Fig. 5a), the plentiful hydroxyl groups on the wood surface absorbed and reacted with the complexes of (Zn[OH]_4_)^2−^ (Fig. 5b). Due to the similar hydrophilic properties of wood and the precursors of ZnO in nature, the complexes of (Zn[OH]_4_)^2−^ preferentially react with the hydroxyl groups of the wood surface than with the FAS molecules through a combination of hydrothermal particle formation and hydrogen-bond-driven surface modification^27^,^28^. After that, (Zn[OH]_4_)^2−^ units continue to develop and form ZnO nanorod arrays, which grow on the wood surface in the alkaline atmosphere as the synthesis advances through the interaction of hydroxyls (Fig. 5c,f). Owing to the existence of FAS, the overall energetic balance between entropic and enthalpic contributions has a positive value. Therefore, this path can be considered unviable (towards the left of the condensation reaction in Fig. 5). On the other hand, the condensation of a second silane molecule in a vicinal surface hydroxyl group and the parallel condensation of the two silane molecules releasing two further water molecules (towards the left of condensation reaction in Fig. 5) is a very favourable process (ΔG < 0 kcal/mol). Thus, it is expected that the formation of infinite silane chains grafted on the surface would be a spontaneous process because of both the mentioned entropic gains. Consequently, the Si-OH terminal groups of FAS are attached onto the surface of ZnO nanorod arrays through hydrogen bonds via a self-assembly process^29^,^30^. Subsequently, with the continuous dehydration reaction, Si-O groups with superamphiphobic long-chain alkyl groups and -fluorocarbon groups are formed on the surfaces of ZnO nanord arrays and wood (Fig. 5d,g). Finally, the as-obtained wood sample demonstrates a preferable superamphiphobic capability (Fig. 5e).

**Figure 6:**
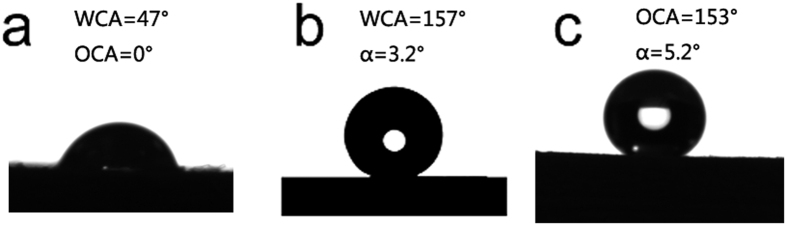
(**a**) WCA and OCA of the pristine wood; CAs and roll-off angles of ZNA-treated wood, (**b**) water and (**c**) hexadecane.

The wettability of the pristine wood and ZNA-treated wood were characterized by measuring the CAs for both water and hexadecane, as shown in Fig. 6. In Fig. 6a, the WCA and the OCA for the pristine wood were 47° and 0°, respectively, which demonstrated that the unmodified wood surface was hydrophilic and totally oleophilic. However, after the one-step solvothermal treatment, the ZNA-treated wood exhibited both superhydrophobicity with a WCA of 157° and a roll-off angle (α) of 3.2° (Fig. 6b) and superoleophilicity with an OCA of 153° and a roll-off angle (α) of 5.2° (Fig. 6c). Generally speaking, a surface showing CA more than 150° and α less than 10° will show super-liquid-repellent properties^31^,^32^. The results show that the hydrophilic surface of the pristine wood has been successfully transformed into a superamphiphobic surface after the solvothermal treatment. This might be because the high surface concentration of –CH_2_, –CH_3_, –CF_2_ and –CF_3_ groups, which provide the low surface energy required to achieve superamphiphobicity^33^,^34^.

**Figure 7:**
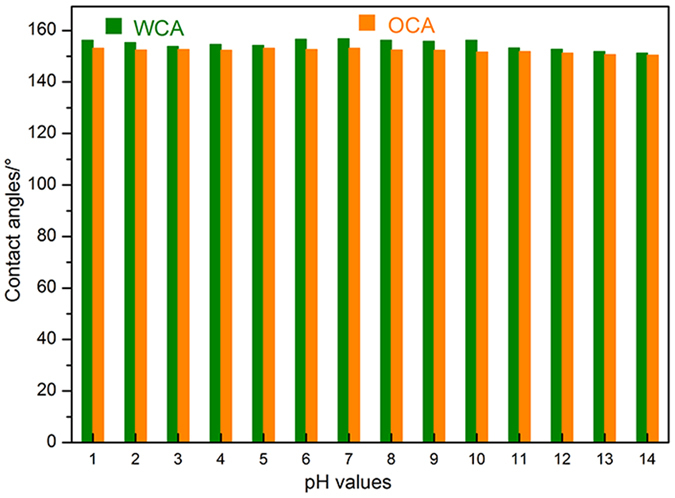
The relationship between pH values and the contact angles (WCA and OCA) of the ZNA-treated wood.

It is well-known that wood or wood-based materials are prone to erode under wet conditions, especially in corrosive surroundings. To assess the robust superamphiphobic performance of the prepared ZNA-treated wood samples, they were immersed into solutions with different pH values, adjusted using HCl or NaOH, for 100 h. The experimental measurements show that the obtained wood surface always exhibited superamphiphobicity in the entire range of studied pH values from 1 to 14 (Fig. 7). Interestingly, the ZNA-treated wood seemed to be more superamphiphobic when subjected to acid corrosive solutions than that to alkaline corrosive solutions. This might be due to the decomposition of FAS-17, which provided a high surface concentration of −CF_3_ and −CF_2_ as a barrier, effectively protecting ZnO nanostructures from acid and other degradations. Thus, the ZNA-treated wood with such a film showed a strong anti-acid capacity, and the as-prepared surface had an anticorrosive property for many corrosive liquids. This conclusion has important implications for anticorrosive wood materials as engineering materials because superamphiphobicity to corrosive liquids could greatly extend the application of wood materials in many industrial fields, especially in harsh conditions.

**Figure 8:**
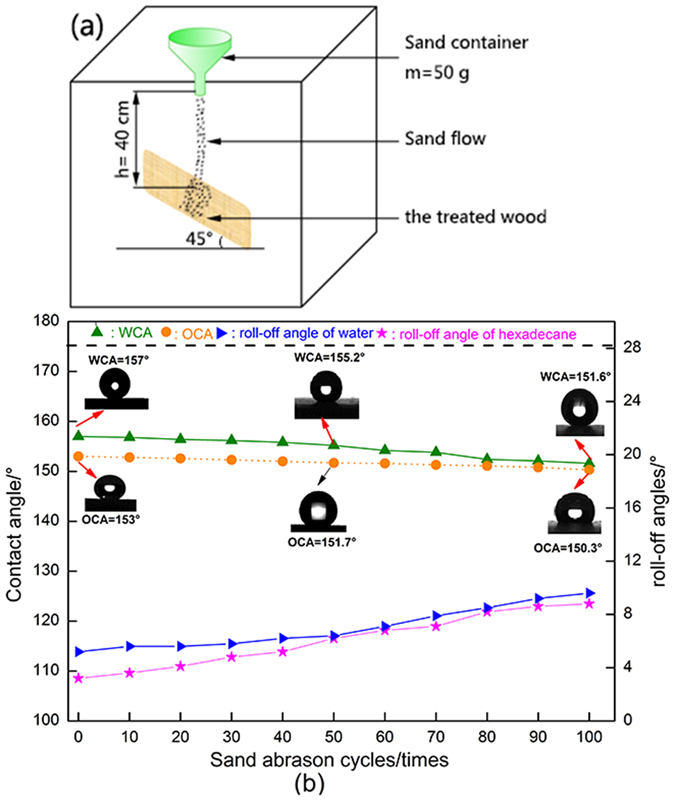
Robust superamphiphobic performances of the ZNA-treated wood evaluated by a sand abrasion test. (**a**) Schematic illustration of the sand abrasion test. (**b**) WCAs, OCAs, roll-off angles of water, and roll-off angle of hexadecane varied with the corresponding sand abrasion cycles.

The robust superamphiphobic performance of the ZNA-treated wood was further mechanically evaluated by sand abrasion tests, as shown in Fig. 8. In Fig. 8a, used sand grains with an average diameter of 200 μm were dropped on a ZNA-treated wood surface from a height of 40 cm. The superamphiphobic wood surface was repeatedly impacted by the sand grains 100 times. The contact angles (WCA and OCA) and roll-off angles were recorded after every ten times. The observations in Fig. 8b clearly show that the WCAs as well as OCAs gradually decreased with an increasing number of cycles of sand impacts as well as increasing roll-off angles. However, the ZNA-treated wood still remained superhydrophobic, with a WCA of 151.6°, and superoleophobic, with an OCA of 150.3° after 100 cycles of sand abrasion tests. The simulated sand abrasion performance implies that the as-prepared superamphiphobic wood surface produced via the one-step solvothermal method possesses robust mechanical stability and durability to external damage impact. These materials might be greatly appreciated by a more discerning and demanding consumer market as high-value-added products.

**Figure 9:**
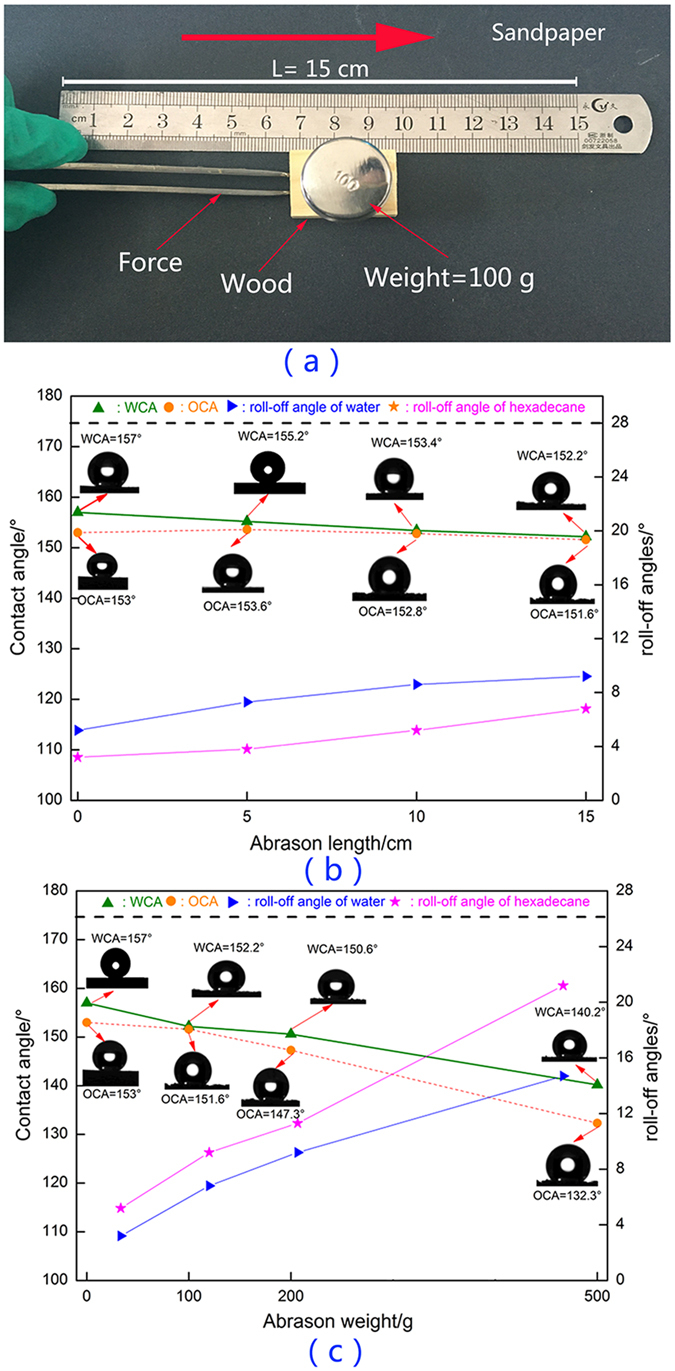
Sandpaper abrasion test. (**a**) Schematic illustration of the sandpaper abrasion test. (**b**) The effect of sandpaper abrasion length and (**c**) abrasion weight on the WCAs, OCAs, roll-off angles of water, and roll-off angle of hexadecane at a certain abrasion length.

To further investigate the mechanical stability of the robust superamphiphobic performance of the ZNA-treated wood, two complementary sandpaper abrasion tests were carried out, as shown in Fig. 9. As shown in Fig. 9a, sandpaper abrasion tests were carried out on the surface of the ZNA-treated wood. A wood surface weighing 100 g was placed face-down on sandpaper (standard sandpaper, grit no. 320) and moved 15 cm along the ruler. The WCAs, OCAs, roll-off angle of water and roll-off angle of hexadecane were measured at a certain abrasion length. Figure 9b clearly shows that the WCAs, as well as OCAs, gradually decreased with increasing length of sandpaper abrasion, as well as the increase of roll-off angles. After one cycle of abrasion up to a length of 15 cm, the ZNA-treated wood still remained superhydrophobic, with a WCA of 155.2° and roll-off angle of 9.2°, but presented decreased superoleophobicity, with an OCA of 151.6° and roll-off angle of 11.3°. As shown in Fig. 9c, wood surfaces with different applied weights (0 g, 100 g, 200 g, and 500 g) were placed face-down on sandpaper and moved for 15 cm along the ruler. It was apparent that the wood surface maintained its superhydrophobic performance in the range from 0 g to 200 g but lost its superoleophobicity when the weight increased to 200 g (the OCA value is 1147.3° and roll-off angle of is 11.3°). When the weight on the wood surface was increased to 500 g, the wood surface totally lost both superhydrophobicity and superoleophobicity. The sandpaper abrasion tests indicated that the as-prepared superamphiphobic wood surface had some limitations for uploading weights. Overloading damaged the superamphiphobic wood surface.

**Figure 10:**
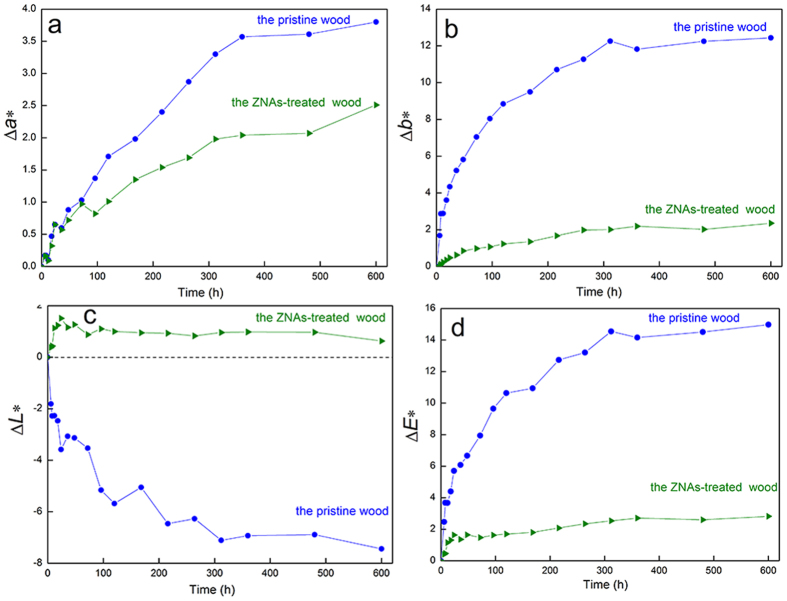
Change tendency of Δa*, Δb*, ΔL*, and ΔE* of the pristine original wood and ZnO covered wood.

To evaluate ultraviolet resistance of the wood sample, surface colour changes were measured for the pristine wood and ZNA-treated wood through an artificially accelerated ageing test. The experimental results are shown in Fig. 10. As shown in Fig. 10a, the Δa* value of the pristine wood turned to red, meaning that the surface colour of the pristine wood became gradually redder with increasing ultraviolet irradiation time. The Δa* values of the ZNA-treated wood showed a similarly changing tendency compared with the pristine wood. However, the Δa* values of the ZNA-treated wood were much smaller (approximately 1/2) than those of the pristine wood, indicating much stronger ultraviolet resistance of the ZNA-treated wood. As shown in Fig. 10b, the changes in Δb* values imply that the surface colour of the pristine wood and ZNA-treated wood turns slightly yellow and then to a dark yellow with increasing ultraviolet irradiation time. As shown in Fig. 10c, the ΔL* value of the pristine wood became negative with increasing ultraviolet irradiation time, suggesting that the lightness of the wood surface appeared to turn black. However, for the ZNA-treated wood, the ΔL* values still remained positive, indicating that the lightness turned to somewhat white. More importantly, the tendency of change of ΔL* for the ZNA-treated wood was slightly different compared to the pristine wood, implying that the lightness of the as-synthesised wood changed only slightly under ultraviolet irradiation, showing a superior ultraviolet-resistant capability. The total colour change (ΔE*) is presented in Fig. 10d. It can be obviously seen that ΔE* values of the pristine wood underwent a dramatic variation with increasing ultraviolet irradiation time, while only a slight change occurred for the ZNA-treated wood. This further confirms the superior ultraviolet resistance of the ZNA-treated wood.

**Figure 11:**
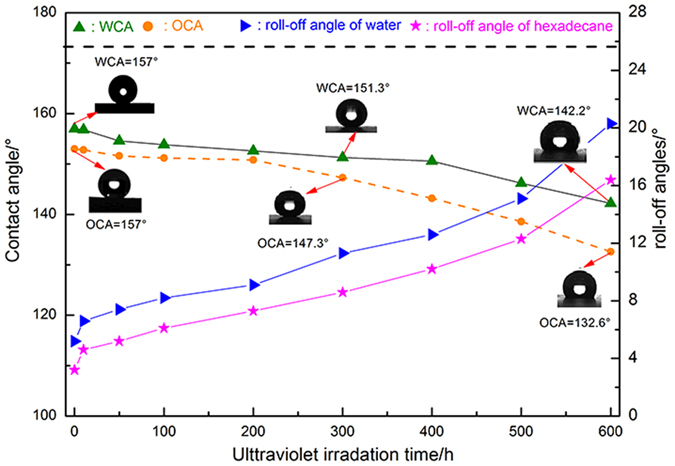
Robust superamphiphobic performances of the ZNA-treated wood evaluated by an artificially accelerated ageing: WCAs, OCAs, roll-off angles of water, and roll-off angles of hexadecane varied with the corresponding ultraviolet time.

In the artificially accelerated ageing tests, the wood sample was subjected not only to ultraviolet irradiation but also to temperature and humidity. Therefore, it seems to be necessary to evaluate the wetting property of the ZNA-treated wood, which would be greatly important for the outdoor wood applications. Figure 11 shows the measured WCA, OCA, roll-off angle of water, and roll-off angle of hexadecane at certain artificially accelerated ageing times. It is obvious whether that the WCAs, as well as OCAs, gradually decreased with increasing ultraviolet irradation time, accompanied with the increase of roll-off angles. In the duration from 0 h to 200 h, the ZNA-treated wood always maintained its superamphiphobicity with a WCA of 152.6°, OCA of 150.8°, and roll-off angles for water and hexadecane of 7.3° or 9.1°, respectively. After 300 h, the ZNA-treated wood still remained superhydrophobic, with a WCA of 152.6° and roll-off angle of 8.6°, but lost superoleophobicity, with an OCA of 147.3° and roll-off angle of 11.3°. After this point, the superamphiphobicity deteriorated and finally, completely vanished by the end of the artificially accelerated ageing test. However, it was noted that the as-prepared wood sample always maintained its amphiphobic performance, even under ultraviolet irradiation, intermittent and transpositional temperatures of 65 °C and 45 °C, and water spray. These results indicate that the ZNA-treated wood prepared by the one-step solvothermal method would be ideal for the outdoor wood applications due to its self-cleaning property and scouring off of dust during rains. To some extent, these obtained results confirm that the ZNA-treated wood has a relatively robust amphiphobic stability and durability.

## Conclusion

In summary, a robust, superamphiphobic ZNA-treated wood was successfully fabricated through a simple, one-step solvothermal method. The superamphiphobic performances were maintained under corrosive liquids, mechanical impact, intermittent and transpositional temperatures, and water spray. Additionally, the as-prepared wood sample showed superior ultraviolet resistance. This work potentially provides a feasible pathway to fabricate ZnO/wood hybrid materials with desirable functions.

The as-fabricated robust superamphiphobic ZNA-treated wood has great potential for anti-corrosion, self-cleaning and high-temperature and -humidity functionalities. However, the presently proposed method has its limitations, presumably related to the stability of the grown ZnO nanostructures and wood dimensional stability. Additionally, environmental impact, costs and safety are challenges for employing autoclaves with enlarged dimensions due to the high temperatures and pressures applied.

## Materials and Methods

### Materials

All the chemicals were supplied by Shanghai Boyle Chemical Co. Ltd. Tangential sections of Poplar Wood (*Populus ussuriensis Kom*) slices with a size of 30 mm (length) × 10 mm (width) × 5 mm (height) were ultrasonically cleaned in deionized water and acetone for 30 minutes and then vacuum-dried at 80 °C for 24 h.

### One-step simple synthesis of ZnO nanorod arrays on a wood surface

In a typical synthesis, 2.2 g of zinc acetate dihydrate, 1.45 g of cetyltrimethylammonium bromide (CTAB) and 1.2 g of sodium hydroxide were mixed in 100 mL of 95% ethanol solution under gentle stirring for 2 h at room temperature. The wood samples were also immersed in the solution. During the stirring process, 1 mL of fluoroalkylsilane was added dropwise into the mixed solution. The full name of FAS is 1H,1H,2H,2H-perfluorodecyltriethoxysilane. It was abbreviated as FAS-17. It has a long perfluoro chain. Following the FAS addition, the solution and wood sample were both transferred into a Teflon-lined autoclave. The autoclave was then sealed and heated at 90 °C for 4 h. The wood samples were then removed and rinsed in deionized water and absolute ethyl alcohol several times. Finally, the samples were dried in an oven at 45 °C over 24 h.

### Characterizations

The morphology of the ZNAs grown on the wood surface was observed by field-emission scanning electron microscopy (FE-SEM, FEI Sirion 200) and transmission electron microscopy (TEM, FEI Tecnai F20). Surface chemical compositions of samples were determined by energy disperse X-ray analysis (EDS).The crystalline structures of ZNAs were identified by X-ray diffraction (XRD, Rigaku, D/MAX 2200), with Cu Kα radiation (λ = 1.5418 Å) at a scan rate (2θ) of 4°/min with an accelerating voltage of 40 kV and an applied current of 30 mA, ranging from 5° to 80°. The chemical changes of the sample were recorded by Fourier transform infrared spectroscopy (FTIR, Magna-IR 560, Nicolet). The thermal performances of the sample were examined in the range from 20 to 700 °C using a thermal analyser (TGA, SDT Q600) at a heating rate of 20 °C min^−1^ and under an N_2_ flow rate of 50 mL·min^−1^. The contact angles (CA) and roll-off angles of water and hexadecane were measured on an OCA40 contact angle system (Dataphysics, Germany) at ambient temperature. The time between the placement of the droplet and the CA measurement was 10 seconds. An average of five measurements taken at different positions on each sample was used to calculate the final CA value.

### Artificially accelerated ageing test

An artificially accelerated ageing test, used to induce photo-discolouration of the samples, was conducted using an ultraviolet accelerated ageing tester (Atlas, Chicago, Illinois, USA), which allowed for water spray, as well as condensation. First, the samples were fixed in stainless steel holders and rotated. Then, the samples were subjected to accelerated ageing by exposure to ultraviolet light radiation at a wavelength of 340 nm and temperature of 60 °C for 2.5 h, followed by a spray of water for 0.5 h, followed by condensation at 45 °C for 24 h. The average irradiance was set to 0.85 W/m^2^ and the temperature of the spray was set to 25 °C. The exposure time ranged from 0 to 600 hour. The measurements for colour change were taken at regular intervals of time. The colour of all the samples was measured before and after ultraviolet irradiation, using a portable spectrophotometer (NF-333, Nippon Denshoku Company, Japan) with a CIELAB system, in accordance with the ISO-2470 standard. The CIELAB system was characterized by three parameters, L*, a*, and b*. The L* axis represents the lightness, which varies from 100 (white) to 0 (black); a* and b* are the chromaticity indices; +a* is the red direction; −a* is green; +b* is yellow; and −b* is blue. The changes of L*, a*, and b* are calculated according to the following equations (1), (2) and (3).


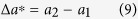



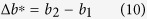



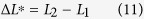


where ΔL*, Δa*, and Δb* are the differences of initial and final values of a*, b*, and L*; *a*_1_, *b*_1_, and *L*_1_ are the initial colour parameters and *a*_2_, *b*_2_, and *L*_2_ are the colour parameters after ultraviolet irradiation.

The overall colour changes (ΔE*) were used to evaluate the total colour change using the following equation (4). A lower ΔE* value corresponds to a lower colour difference and indicates strong resistance to ultraviolet irradiation.


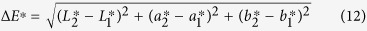


CIE-L*, a*, b* and Δ*E* were measured at five locations of each sample and the average values were calculated as the final decision^35^.

## Additional Information

**How to cite this article**: Yao, Q. *et al*. One-step solvothermal deposition of ZnO nanorod arrays on a wood surface for robust superamphiphobic performance and superior ultraviolet resistance. *Sci. Rep*. **6**, 35505; doi: 10.1038/srep35505 (2016).
